# A marginalized variational bayesian approach to the analysis of array data

**DOI:** 10.1186/1753-6561-2-s4-s7

**Published:** 2008-12-17

**Authors:** Yiming Ying, Peng Li, Colin Campbell

**Affiliations:** 1 Department of Engineering Mathematics, University of Bristol, Bristol BS8 1TR, UK

## Abstract

**Background:**

Bayesian unsupervised learning methods have many applications in the analysis of biological data. For example, for the cancer expression array datasets presented in this study, they can be used to resolve possible disease subtypes and to indicate statistically significant dysregulated genes within these subtypes.

**Results:**

In this paper we outline a marginalized variational Bayesian inference method for unsupervised clustering. In this approach latent process variables and model parameters are allowed to be dependent. This is achieved by marginalizing the mixing Dirichlet variables and then performing inference in the reduced variable space. An iterative update procedure is proposed.

**Conclusion:**

Theoretically and experimentally we show that the proposed algorithm gives a much better free-energy lower bound than a standard variational Bayesian approach. The algorithm is computationally efficient and its performance is demonstrated on two expression array data sets.

## Background

Unsupervised clustering methods from machine learning are very appropriate in extracting structure from biological data sets. There has been extensive work in this direction using hierarchical clustering analysis [1], *K*-Means clustering [2] and self-organizing maps [3]. Bayesian methods are an effective alternative since they provide a mechanism for inferring the number of clusters. They can easily incorporate priors which penalise over-complexed models which would fit to noise and they allow probabilistic confidence measures for cluster membership. In this paper, we focus on Bayesian models which use Dirichlet priors. Examples of these models include Latent Dirichlet Allocation [4] (LDA) for use in text modeling and Latent Process Decomposition (LPD) [5] for analysis of microarray gene expression datasets. One appealing feature of the latter models is that each data point can be stochastically associated with multiple clusters. One approach to model inference is to use methods such as Markov Chain Monte Carlo and Gibbs sampling. However, for the large datasets which occur in many biomedical applications these methods can be too slow for certain tasks such as model selection. This motivates our interest in computationally efficient variational inference methods [4-6].

Typically, these inference methods posit that all the latent variables and model parameters are *independent *of each other (i.e. a fully factorized family) which is a strong assumption. In this paper we propose and study an alternative inference method for LPD, which we call marginalized variational Bayesian (MVB). In this approach the latent process (cluster) variables and model parameters are allowed to be *dependent *on each other. As we will show in the next section, this assumption is made feasible by marginalizing the mixing Dirichlet variables, and then performing inference in the reduced variable space. This new approach to constructing an LPD model theoretically and experimentally provides much better free-energy lower bounds than standard a variational Bayes (VB) approach [6,7]. Moreover, the algorithm is computationally efficient and converges faster, as we demonstrate with experiments using expression array datasets.

## Methods

### The LPD probabilistic model

We start by recalling LPD [5]. Let *d *index samples, *g *the genes (attributes) and *k *the soft clusters (samples are represented as combinatorial mixtures over clusters). The numbers of clusters, genes and samples are denoted K, G, and D respectively. For each data *E*_*d*_, we have a multiple process (cluster) latent variable *Z*_*d *_= {*Z*_*dg*_: *g *= 1,..., G} where each *Z*_*dg *_is a K-dimensional unit-basis vector, i.e., choosing cluster *k *is represented by *Z*_*dg*, *k *_= 1 and *Z*_*dg*, *j *_= 0 for *j *≠ *k*, otherwise. Given the mixing coefficient *θ*_*d*_, the conditional distribution of *Z*_*d *_is given by $p(Z_{d}|\theta_{d})=\prod_{g,k}\theta_{dk}^{Z_{dg,k}}$. The conditional distributions, given the latent variables, is given by $p(E_{d}|Z_{d},\mu ,\beta )=\prod_{g,k}{\left[N(E_{dg}|\mu_{gk},\beta_{gk})\right]}^{Z_{dg,k}}$, where N is the Gaussian distribution with mean *μ *and precision *β*.

Now we introduce conjugate priors over parameters *θ*, *μ*, *β*. Specifically, we choose *p*(*θ*_*d*_) = Dir(*θ*_*d*_|*α*), and $p(\mu )\sim \prod_{g,k}N(\mu_{gk}|m_{0},v_{0})$, and *p*(*β*) distributed as ∏_*gk *_Γ(*β*_*gk*_|*a*_0_, *b*_0_) where Γ is defined by $\Gamma (x|a_{0},b_{0})=x^{a_{0}-1}\exp\left(-\frac{x}{b}\right)/b_{0}^{a_{0}}\Gamma (b_{0})$. We assume the data is i.i.d. and let Θ = {*μ*, *β *}. The joint distribution is given by

(1) $$p(E,\theta ,Z|\Theta )=\prod_{d}p(\theta_{d})p(Z_{d}|\theta_{d})p(E_{d}|\mu ,\beta ,Z_{d}),$$

One can easily see that the marginal likelihood *p*(*E*|Θ) is the same as that in [5]. It is important to note that, in standard Gaussian mixture models [8], each data point is only related with a K-dimensional latent variable which restricts the data to being in one cluster. Instead, in LPD each data point *E*_*d *_is associated with multiple latent variables *Z*_*d *_= {*Z*_*dg*_: *g *= 1,..., G}, and thus *E*_*d *_is stochastically associated with multiple clusters.

### Marginalized variational Bayes

In this section we describe a marginalized variational Bayesian approach for LPD. The target of model inference is to compute the posterior distribution *p*(*θ*, *Z*, Θ |*E*) = *p*(*E*, *θ*, *Z*|Θ)*p*(Θ)/*p*(*E*). Unfortunately, this involves computationally intensive estimation of the integral in the evidence *p*(*E*). Hence, we approximate the posterior distribution in a *hypothesis family *whose element are denoted by *q*(*θ*, *Z*, Θ).

The standard variational bayesian method [7,10] uses the equality:

(2) $$\begin{array}{c} \log p(E)=\log\int \sum_{Z}p(E,\theta ,Z,\Theta )d\theta d\Theta \\ =E_{q}\left[\log\frac{p(E,\theta ,Z|\Theta )p(\Theta )}{q(\theta ,Z,\Theta )}\right]+\text{KL}(q(\theta ,Z,\Theta )||p(\theta ,Z,\Theta ))). \end{array}$$

Our optimization target is to maximize the free-energy: $E_{q}\left[\log\frac{p(E,\theta ,Z|\Theta )p(\Theta )}{q(\theta ,Z,\Theta )}\right]$ which, equivalently, minimizes the KL-divergence. One standard way is to choose the hypothesis family in a factorized form *q*(*θ*, *Z*, Θ) = *q*(*θ*)*q*(*Z*)*q*(Θ). In this setting, the free-energy lower bound (2) for the likelihood can be written as:

(3) $$ℒ(q(\theta ),q(Z),q(\Theta )):=E_{q}\left[\log\frac{p(E,\theta ,Z|\Theta )}{q(\theta ),q(Z)}\right]-\text{KL}(q(\Theta ||p(\Theta ))).$$

In this paper we study an alternative approach motivated by [9] which only marginalizes the latent variable *θ *and do variational inference only with respect to the leftover latent variable *Z*. In essence, we assume that the latent variables *θ *can be dependent on *Z*, Θ and the hypothesis family is chosen in the form of *q*(*θ*, *Z*, Θ) = *q*(*θ *|*Z*, Θ)*q*(*Z*)*q*(Θ). Since the distribution *q*(*θ*|*Z*, Θ) is arbitrary, let it be equal to $p(\theta |E,Z,\Theta )=\frac{p(E,\theta ,Z,\Theta )}{p(E,Z,\Theta )}$. Putting this into equation (2) and observing that $\frac{p(E,\theta ,Z|\Theta )}{p(\theta |E,Z,\Theta )}p(E,Z|\Theta )$ gives

(4) $$\log p(E)=E_{q}[\log\frac{p(E,Z|\Theta )}{q(Z)}]-\text{KL}(q(\Theta ||p(\Theta )))+\text{KL}(p(\theta |Z,\Theta )q(\Theta )q(Z)||p(\theta ,Z,\Theta ))$$

(5) $$=E_{q}[\log\frac{p(E,Z|\Theta )}{q(Z)}]-\text{KL}(q(\Theta ||p(\Theta )))+\text{KL}(q(Z)q(\Theta )||p(Z,\Theta )).$$

Therefore, it is sufficient to maximize the lower bound

(6) $$ℒ(q(Z),q(\Theta ):=E_{q(\Theta )q(Z)}\left[\log\frac{p(E,Z|\Theta )}{q(Z)}\right]-\text{KL}(q(\Theta ||q(\Theta )).$$

Observe that log $\frac{p(E,Z|\Theta )}{q(Z)}\geq \int q(\theta )\log\frac{p(E,\theta ,Z|\Theta )}{q(Z)q(\theta )}d\theta$. Consequently, we see that

(7) ℒ(q(θ),q(Z),q(Θ))≤ℒ(q(Z),q(Θ)).

As mentioned above, since *θ *can be dependent on *Z*, Θ, marginalized VB (MVB) yields a tighter lower bound for the likelihood than the standard VB approach in [6], thus potentially yielding better clustering results.

### Model inference and learning

We now turn our attention to the derivation of updates for marginalized VB following the inference methodology [7,10]. For simplicity, let the posterior distribution *q*(*Z*), *q*(*μ*), *q*(*β*) be indexed by parameters. Specifically, we assume that $q(Z)=\prod_{d,g,k}r_{dg,k}^{Z_{dg,k}}$, $q(\mu )=\prod_{g,k}N(\mu_{gk}|m_{gk},v_{gk})$, and *q*(*β*) = ∏_*g*, *k *_Γ(*β*_*gk*_|*a*_*gk*_, *b*_*gk*_). Correspondingly, the free-energy lower bound ℒ(*q*(*Z*), *q*(Θ)) in equation (6) becomes a variational functional over these parameters, and hence we use ℒ(*R*, *μ*, *β*) later on. The model inference can be summarized by the following coordinate ascent updates.

Let *Z*^\*dg *^denote the random variables excluding *Z*_*dg*_. For any *d*, *g *let Θ and *Z*^\*dg *^be fixed, then we take the functional derivative of the free-energy ℒ(*q*(*Z*), *q*(Θ)) w.r.t. *q*(*Z*_*dg*_) and obtain the update:

(8) $$q(Z_{dg})\propto \exp\left(E_{q^{\backslash dg}}[\log p(E,Z|\Theta )]\right).$$

For the updates for *q*(Θ), we obtain

(9) $$q(\mu )\propto p(\mu )\exp\left(E_{q^{\backslash \mu }}[\log p(E,Z|\Theta )]\right),q\left(\beta \right)\propto p\left(\beta \right)\exp\left(E_{q^{\backslash \beta }}[\log p(E,Z|\Theta )]\right).$$

Marginalizing out *θ *in (1) yields

(10) $$\begin{array}{l} p(E,Z|\Theta ):=\prod_{d}\left[p(Z_{d}|\alpha )\right]p(E_{d}|\mu_{d},\beta_{d},Z_{d})\\ =\prod_{d}\left[\frac{\Gamma (\sum_{k}\alpha_{k})}{\Gamma (\sum_{k}\alpha_{k}+\sum_{g,k}Z_{dg,k})}\prod_{k}\frac{\Gamma (\alpha_{k}+\sum_{g}Z_{dg,k})}{\Gamma (\alpha_{k})}\right]\prod_{g,k}{\left[N\left(E_{dg}|\mu_{gk},\beta_{gk}\right)\right]}^{Z_{dg,k}}. \end{array}$$

Estimating the expectations of the log likelihoods in equations (8) and (9), we derive the variational EM-updates as follows. Details are postponed to the Appendix.

**E-step**: using equation (8) and denoting the digamma function by *ψ*, we have

(11) $$r_{dg,k}\propto \frac{\left(\alpha_{k}+\sum_{g^{\prime }\neq g}r_{dg^{\prime },k})\exp(N_{dg,k}\right)}{\exp\left(\frac{\sum_{g^{\prime }\neq g}r_{dg^{\prime },k}(1-r_{dg^{\prime },k})}{2{\left(\alpha_{k}+\sum_{g^{\prime }\neq g}r_{dg^{\prime },k}\right)}^{2}}\right)}$$

where *N*_*dg*, *k *_is given by 0.5(*ψ*(*a*_*gk*_)+log *b*_*gk*_) - 0.5*a*_*gk*_*b*_*gk*_((*E*_*dg *_- *m*_*gk*_)^2 ^+ $v_{gk}^{-1}$) and *r*_*dg*, *k *_should be normalized to one over *k*.

**M-step**: using equation (9):

(12) $$v_{gk}=v_{0}+a_{gk}b_{gk}\sum_{d}r_{dg,k},$$

(13) $$m_{gk}=\frac{1}{v_{gk}}[v_{0}m_{0}+a_{gk}b_{gk}\sum_{d}r_{dg,k}E_{dg}],$$

(14) $$a_{gk}=a_{0}+0.5\sum_{d}r_{dg,k},$$

(15) $$\frac{1}{b_{gk}}=\frac{1}{b_{0}}+0.5\sum_{d}r_{dg,k}\left[{\left(E_{dg}-m_{gk}\right)}^{2}+\frac{1}{v_{gk}}\right].$$

We pursue the above iterative procedure until convergence of the lower bound ℒ(*R*; Θ) whose evaluation is given in the Appendix. Since *Z*_*dg*, *k *_determines the cluster for the observed data point *E*_*d *_at attribute *g *and *r*_*dg*, *k *_is its expectation, we intuitively assign data *E*_*d *_to cluster arg max{∑_*g*_*r*_*dg*;*k*_: *k *= 1,...., K}. We can also do model selection over the number of clusters based on a free energy lower bound of the marginalized VB. Experiments in the next section show that this approach is reasonable.

## Results

We ran marginalized VB on three data sets. The first was the wine data set from the UCI Repository [11]: this has 178 samples and each sample has 13 features. This data set was chosen for the purpose of validating the proposed method since there are 3 distinct clusters present (derived from 3 cultivars). As more biologically relevant examples we then selected two cancer expression array datasets. The first of these was a lung cancer data set [12] consisting of 73 samples and 918 features. The second was a leukemia data set [13] with 90 samples and 500 features. All the data sets were normalized to zero mean and unit variance and the hyper-parameters *m*_0_, *v*_0_, *a*_0_, and *b*_0 _were chosen to have the same values in both standard VB and marginalized VB. Since the datasets are normalized and *m*_0_, *v*_0 _are hyper-parameters of the Gaussian prior distribution over the mean for the data, it is reasonable to choose *m*_0 _= 0, *v*_0 _= 1. For similar reasons, given *a*_0_, *b*_0 _are hyper-parameters of the Gamma prior distribution over the precision (inverse variance) of the data and the mean of a Gamma distributed random variable is *a*_0_*b*_0_, we chose *a*_0 _= 20 and *b*_0 _= 0.05 throughout these experiments.

**Figure 1:**
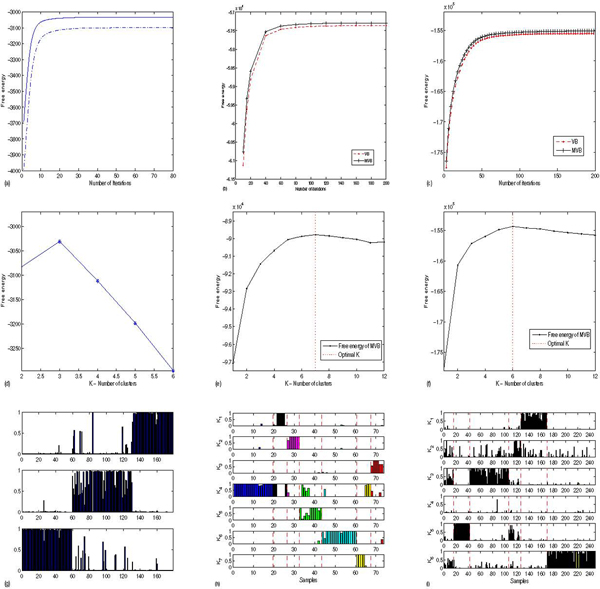
Results for the wine data set (left column), lung cancer data set (middle column) and leukemia data set (right column). Top row (a-c): free energy bounds comparison (upper curve:MVB, lower curve:VB). Middle row (d-f): free energy (*y*-axis) versus K, the number of clusters. Bottom row (g-i): the normalized ∑_*g*_*r*_*dg*, *k *_gives a confidence measure that sample *d *belongs to a cluster *k*. For the two cancer datasets, samples separated by dashed lines belong to an identified class e.g. adenocarcinoma samples or small cell lung cancer samples (figure (h), see text).

First we compared the free energy lower bound of marginalized VB and standard VB based on 30 random initialization. In Figure 1 (top row) we observe an improvement in the free energy as a function of iteration step, for marginalized VB over standard VB. In analogy to standard VB, marginalized VB can determine the appropriate number of soft clusters by estimating the free energy bound given by equation (6) in contrast to the hold-out cross-validation procedure for a maximum likelihood approach to LPD [5]. To investigate the effectiveness of this approach to model selection, free energies were averaged over 20 runs from different random initializations. As shown in Figure 1 (middle row), marginalized VB performed well in determining the correct number of clusters (three) in the UCI wine data set. For the cancer array datasets, the peak in the averaged free energy is less marked with an indication of six soft clusters for the leukemia data set and seven clusters for the lung cancer data set.

In the bottom row of Figure 1, we see that marginalized VB shows quite promising clustering results using the normalized ∑_*g*_*r*_*dg*, *k*_: these peaks indicate the confidence in the allocation of the *d*th sample to the *k*th cluster and accord well with known classifications. The lung cancer dataset of Garber *et al *[12] (middle column, Figure 1) consisted of 73 gene expression profiles from normal and tumour samples with the tumours labelled as squamous, large cell, small cell and adenocarcinoma. The samples are in the order in which they are presented in the original paper [12] with the dashed lines showing their original principal sample groupings. As with Garber *et al *[12] we identified seven clusters in the data with the adenocarcinoma samples falling into three separate clusters with strong correlation with clinical outcomes. For their ordering (which we follow) samples 1–19 belong to adenocarcinoma cluster 1, samples 20–26 belong to adenocarcinoma cluster 2, samples 27–32 are normal tissue samples, samples 33–43 are adenocarcinoma cluster 3, samples 44–60 are squamous cell carcinomas, samples 61–67 are small cell carcinomas and samples 68–73 are from large cell tumours.

As our last example, we applied the proposed MVB method to an oligonucleotide microarray dataset from 360 patients with acute lymphoblastic leukemia (ALL) from Yeoh *et al *[13]. ALL is known to have a number of subtypes with variable responses to chemotherapy. In many cases fusion genes are implicated in the genesis of the disease. For the Yeoh *et al *[13] dataset, samples were drawn from leukemias with rearrangements involving *BCR-ABL*, *E2A-PBX1*, *TEL-AML1*, rearrangements of MLL gene, hyperdiploid karyotope (more than 50 chromosomes) and T lineage leukemias (*T-ALL*). The free energy is plotted in Figure 1 (right column, middle row) with a peak suggesting 6 subtypes. The dashed lines represent the original demarcations of groups according to known genetic rearrangement. Samples 1–15 are *BCR-ABL*, 16–42 are *E2A-PBX1*, 43–106 *Hyperdiploid *> 50, 107–126 *MLL*, 206–248 *T-ALL*, 249–327 *TEL-AML1*, 328–335 *Group23 *and 127–205 are labelled as *Others*. Some groupings, such as *E2A-PBX1*, are very distinct. However, the overall groupings are not as well defined as with lung cancer.

## Conclusion

We have proposed an efficient variational Bayesian inference method for LPD probabilistic models. By allowing the variables to be dependent on each other, the method can provide more accurate approximation than standard VB. Also, the method provides a principled approach to model selection via the free energy bound. Promising clustering results were also reported on lung cancer and leukemia data sets. Extensions of this method to semi-supervised clustering will be reported elsewhere.

## Competing interests

The authors declare that they have no competing interests.

## Authors' contributions

YY and CC conceived the method and drafted the manuscript. PL and YY coded the algorithm and implemented the experiments. All authors read and approved the final manuscript.

## Appendix

In this appendix we derive the EM-updates and free energy bound for MVB.

### Derivation of updates

Noting that, for any *d*, *g*, ∑_*k*_*Z*_*dg*, *k *_= 1 and denoting the number of features by G we obtain from equation (10):

(16) $$\begin{array}{l} \log p(E,Z|\Theta )=D\log\Gamma (\sum_{k}\alpha_{k})-D\sum_{k}\log\Gamma (\alpha_{k})-D\log\Gamma (\sum_{k}\alpha_{k}+G)\\ +\sum_{d^{\prime },k}\log\Gamma (\alpha_{k}+\sum_{g^{\prime }}Z_{d^{\prime }g^{\prime },k})+\sum_{d^{\prime },g^{\prime },k}Z_{d^{\prime }g^{\prime },k}\log N(E_{d^{\prime }g^{\prime }}|\mu_{g^{\prime }k},\beta_{g^{\prime }k}). \end{array}$$

Since $\Gamma \left(\alpha_{k}+\sum_{g^{\prime }\neq g}Z_{dg^{\prime },k}+Z_{dg,k}\right)={\left(\alpha_{k}+\sum_{g^{\prime }\neq g}Z_{dg^{\prime },k}\right)}^{Z_{dg,k}}\Gamma \left(\alpha_{k}+\sum_{g^{\prime }\neq g}Z_{dg^{\prime },k}\right)$, putting this observation into the log *p*(*E*, *Z*|Θ) yields:

$$\begin{array}{lll} E_{q\backslash dg}\left(\log p(E,Z|\Theta )\right) & = & \sum_{k}Z_{dg,k}(E_{q\backslash dg}\left[\log\left(\alpha_{k}+\sum_{g^{\prime }\neq g}Z_{dg^{\prime },k}\right)\right]\\ & + & E_{q(\Theta )}\left[\log N(E_{dg}|\mu_{gk},\sigma_{gk})\right])+\text{constant terms}, \end{array}$$

where *constant terms *are independent of *Z*_*dg*, *k*_. Hence, substituting this into equation (8) we conclude that

(17) $$r_{dg,k}\propto \exp\left(E_{q(\Theta )}\left[\log N(E_{dg}|\mu_{gk},\beta_{gk})\right]+\log E_{q\backslash dg}\left[\log\left(\alpha_{k}+\sum_{g^{\prime }\neq g}Z_{dg^{\prime },k}\right)\right]\right).$$

To estimate the expectation of the Normal distribution, we use the following observations (e.g. [7]) for the Gamma and Normal distributions:

$$\begin{array}{cc} E_{q(\beta )}[\beta_{gk}]=a_{gk}b_{gk}, & E_{q(\beta )}[\log\beta_{gk}]=\psi (a_{gk})+\log b_{gk}, \end{array}$$

and

$$E_{q(\mu )}[\mu_{gk}^{2}]=m_{gk}^{2}+v_{gk}^{-1},E_{q(\mu )}[\mu_{gk}]=m_{gk}.$$

Consequently, simple manipulation yields:

$$E_{q(\Theta )}\left[\log N(E_{dg}|\mu_{gk},\beta_{gk})\right]$$

equals, up to a constant term:

(18) $$0.5(\psi (a_{gk})+\log b_{gk})-0.5a_{gk}b_{gk}({\left(E_{dg}-m_{gk}\right)}^{2}+v_{gk}^{-1}).$$

We also use approximating methods [9] to estimate log $E_{q\backslash dg}\left[\log(\alpha_{k}+\sum_{g^{\prime }\neq g}Z_{dg^{\prime },k})\right]$. For this purpose, we observe, for any positive random variable *x*, that

$$E\left(\log(\alpha_{k}+x)\right)\approx \log(\alpha_{k}+Ex)-\frac{\text{Var}(x)}{2{\left(\alpha_{k}+Ex\right)}^{2}},$$

and $E_{q\backslash dg}\left[\sum_{g^{\prime }\neq g}Z_{dg^{\prime },k}\right]=\sum_{g^{\prime }\neq g}r_{dg^{\prime },k}$, $\text{Var}\left(\sum_{g^{\prime }\neq g}Z_{dg^{\prime },k}\right)=\sum_{g^{\prime }\neq g}r_{dg^{\prime },k}(1-r_{dg^{\prime },k})$. Plugging the above observations into equation (17) yields the desired E-step updates.

For the updates for *q*(Θ), the updates are essentially the same as those in [6,7] since the associated terms with variables with Θ in $E_{q\backslash \mu }\left[\log p(E,Z|\Theta )\right]$[log *p*(*E*, *Z*|Θ)] are exact the same, that is, Θ only appears in the Normal distribution. Hence, noting that the product of two Gamma (Normal) distributions is a Gamma (Normal) distribution, we can obtain, from equations (16) and (9), the M-step updates.

### Free energy bound

The free-energy lower bound of marginalized VB is defined by equation (6):

$$ℒ(R;\Theta )=E_{q}[\log p(E,Z|\Theta )]-E_{q(Z)}[q(Z)]-\text{KL}\left(q(\mu )||p(\mu )\right)-\text{KL}\left(q(\beta )||p(\beta )\right).$$

From the fact that Γ(*x *+ 1) = *x*Γ(*x*) for any *x *> 0, we know that $\Gamma (\alpha_{k}+\sum_{g}Z_{dg,k})=\Gamma (\alpha_{k})\prod_{g=1}^{G}{\left(\alpha_{k}+\sum_{j=g+1}^{G}Z_{dj,k}\right)}^{Z_{dg,k}}$, where we use the convention $\sum_{j=G+1}^{G}=0$. Putting this equation into the expression (10) of log likelihood, we obtain:

$$\begin{array}{l} E_{q}[\log p(E,Z|\Theta )]=D\log\Gamma (\sum_{k}\alpha_{k})-D\sum_{k}\log\Gamma (\alpha_{k})-D\log\Gamma (\sum_{k}\alpha_{k}+G)\\ +\sum_{d,k}E_{q}[\log\Gamma (\alpha_{k}+\sum_{g}Z_{dg,k})]+\sum_{d,g,k}r_{dg,k}\log N(E_{dg}|\mu_{gk},\sigma_{gk})\\ =D\log\Gamma (\sum_{k}\alpha_{k})-D\log\Gamma (\sum_{k}\alpha_{k}+G)\\ +\sum_{d,g,k}r_{dg,k}(E_{q}[\log(\alpha_{k}+\sum_{j\geq g+1}Z_{dj,k})]+\log N(E_{dg}|\mu_{gk},\sigma_{gk})). \end{array}$$

Since we used the convention $\sum_{j\geq G+1}Z_{dj,k}=0$, $E_{q}\left[\log\left(\alpha_{k}+\sum_{j\geq G+1}Z_{dj,k}\right)\right]=\log\alpha_{k}$. It remains to estimate the term $E_{q}\left[\log\left(\alpha_{k}+\sum_{j\geq g+1}Z_{dj,k}\right)\right]$ for *g *= 1,..., G - 1. To this end, we use the approximation (18) again to get:

$$E_{q}[\log(\alpha_{k}+\sum_{j\geq g+1}Z_{dj,k})]\approx \log(\alpha_{k}+\sum_{j\geq g+1}r_{dj,k})-\frac{\sum_{j\geq g+1}r_{dj,k}(1-r_{dj,k})}{2{\left(\alpha_{k}+\sum_{j\geq g+1}r_{dj,k}\right)}^{2}}.$$

Consequently, we conclude:

(19) $$\begin{array}{l} E_{q}[\log p(E,Z|\Theta )=D\log\Gamma (\sum_{k}\alpha_{k})-D\log\Gamma (\sum_{k}\alpha_{k}+G)\\ +\sum_{d,g,k}r_{dg,k}[\log(\alpha_{k}+\sum_{j\geq g+1}r_{dj,k})-\frac{\sum_{i\geq g+1}r_{dj,k}(1-r_{dj,k})}{2{\left(\alpha_{k}+\sum_{j\geq g+1}r_{dj,k}\right)}^{2}}]\\ +\sum_{d,g,k}r_{dg,k}[-0.5\log2\pi +0.5(\psi (a_{gk})+\log b_{gk})\\ -0.5a_{gk}b_{gk}(\frac{1}{v_{gk}}+{\left(E_{dg}-m_{gk}\right)}^{2}]. \end{array}$$

where the convention $\sum_{g\geq G+1}^{G}=0$ is used again.

In addition,

$$E_{q(Z)}\left[\log q(Z)\right]=\sum_{d,g,k}r_{dg,k}\log r_{dg,k}.$$

For the KL divergences, we have:

$$\text{KL}\left(q(\mu )||p(\mu )\right)=\sum_{g,k}0.5\log\frac{v_{gk}}{v_{0}}+0.5v_{0}{\left[m_{gk}-m_{0}\right]}^{2}+0.5\left(\frac{v_{0}}{v_{gk}}-1\right),$$

and

$$\begin{array}{lll} \text{KL}(q(\beta ||p(\beta ))) & = & \sum_{g,k}\left(a_{gk}-a_{0})\psi (a_{gk})-\log b_{gk}-a_{gk}-\log\Gamma (a_{gk}\right)\\ & + & \log\Gamma (a_{0})+a_{0}\log b_{0}-(a_{0}-1)(\psi (a_{gk})+\log b_{gk})+\frac{a_{gk}b_{gk}}{b_{0}}. \end{array}$$
